# Hierarchical self-assembly of organic heterostructure nanowires

**DOI:** 10.1038/s41467-019-11731-7

**Published:** 2019-08-26

**Authors:** Ming-Peng Zhuo, Jun-Jie Wu, Xue-Dong Wang, Yi-Chen Tao, Yi Yuan, Liang-Sheng Liao

**Affiliations:** 1 Institute of Functional Nano & Soft Materials (FUNSOM), Jiangsu Key Laboratory for Carbon-Based Functional Materials & Devices, Soochow University, 199 Ren’ai Road, Suzhou, Jiangsu 215123 P. R. China; 2 Institute of Organic Optoelectronics, JITRI, Wujiang, Suzhou, Jiangsu 215211 P. R. China

**Keywords:** Halogen bonding, Molecular self-assembly, Organic molecules in materials science

## Abstract

Organic heterostructures (OHSs) integrating the intrinsic heterostructure characters as well as the organic semiconductor properties have attracted intensive attention in material chemistry. However, the precise bottom-up synthesis of OHSs is still challenging owing to the general occurrence of homogeneous-nucleation and the difficult manipulation of noncovalent interactions. Herein, we present the rational synthesis of the longitudinally/horizontally-epitaxial growth of one-dimensional OHSs including triblock and core/shell nanowires with quantitatively-manipulated microstructure via a hierarchical self-assembly method by regulating the noncovalent interactions: hydrogen bond (−15.66 kcal mol^−1^) > halogen bond (−4.90 kcal mol^−1^) > π-π interaction (−0.09 kcal mol^−1^). In the facet-selective epitaxial growth strategy, the lattice-matching and the surface-interface energy balance respectively facilitate the realization of triblock and core/shell heterostructures. This hierarchical self-assembly approach opens up avenues to the fine synthesis of OHSs. We foresee application possibilities in integrated optoelectronics, such as the nanoscale multiple input/out optical logic gate with high-fidelity signal.

## Abstract (web summary)

Organic heterostructures attract attention in material chemistry but the precise bottom-up synthesis is still challenging. Herein the authors present a hierarchical self-assembly approach to synthesize one-dimensional organic heterostructures by regulating the noncovalent interactions.

## Introduction

Organic heterostructures (OHSs) hold the intrinsic heterostructure characters of rectifying behavior and photovoltaic effect^1,2^, as well as the unique advantages of organic semiconductor molecules, such as the low cost large-area fabrication, tailor-made molecular structure, and compatibility with plastic substrates^3^. In the past several decades, OHSs have attracted particular attention in various organic optoelectronics, including organic photodetectors^4–6^, organic solar cells (OSCs)^7–10^, and organic field-effect transistors (OFETs)^11–13^. The OSCs with the internal donor-acceptor heterojunction comprising poly(2-methoxy-5-(2’-ethyl-hexyloxy)-1,4-phenylenevinylene) (MEH-PPV) and fullerenes achieved higher efficiencies by more than two orders of magnitude than that of the devices made with pure MEH-PPV^14^. However, the simply physical mixing of electron donor/acceptor pairs for the formation of the OHSs usually give rise to the undesirable occurrence of phase separation and leads to poor performance^15–17^.

To meet the practical nanotechnological requirements, controllably manufacturing the OHSs are necessary for high-performance optoelectronics^18–20^. Since the pioneer work by Prof. Aida T. and coworkers, the nanotubes with a coaxial donor-acceptor configuration demonstrate a quick photoconductive response with a large on/off ratio greater than 10^4^, while the complex microfibers exhibit almost no photocurrent generation^21^. Furthermore, the OHSs waveguides can simultaneously exhibit the passive^22–24^ and active^25–28^ modes for the optical modulation and processing, which cannot be achieved in the single-component organic micro-/nanostructure waveguides^25^. Recently, many efforts have been directed toward the fabrication of the OHSs waveguides using laser-induced chemical reactions techniques^22,29^, and micro-manipulation^30^. In comparison, the self-assembled OHSs, such as the branched nanowire heterostructures, inherently exhibit various advantages including low cost, facile fabrication procedures, and lack of mechanical damage^31,32^. Nevertheless, in the self-assembly process, the controlled manipulation for both the diverse weak noncovalent interactions, involving π–π and charge-transfer (CT) interactions, hydrogen/halogen bonds, and van der Walls force^33–35^, and the distinctively different assembly features of organic molecules^36^, remains a largely unsolved problem, leading to a huge challenge to achieve the precise construction of the OHSs. The controlled regulation on the uniform morphological dimensions of the organic nanomaterials has achieved some successes via exploiting the directional noncovalent interactions or introducing other strong interactions^32^, such as strong π-π interaction and hydrogen bond for the self-assembled one-dimensional (1D) nanostructure of aromatic organic molecules^37^. In this regard, it is a feasible pathway to rationally design and synthesize the OHSs by introducing and manipulating multiple dominant noncovalent interactions, which is an undeveloped domain.

Herein, we develop a hierarchical self-assembly approach by tuning the noncovalent interactions for the longitudinally-epitaxial growth (LG) of green-red-green triblock nanowires (TNWs) and the horizontally-epitaxial growth (HG) of core/shell nanowires (C/S-NWs). The lattice-matching and the surface-interface energy balance respectively facilitate the realization of the TNWs via kinetically controlled growth pathway under the stock solution at 25 °C and the C/S-NWs via thermodynamically controlled growth pathway under the stock solution at 55 °C. Moreover, the facile precise modulation in the formation ratio of the hydrogen/halogen bonds can realize the quantitative manipulation for both the length ratio of red-emissive part in the TNWs and the exposed-degree of the core at the center in the C/S-NWs. Significantly, the presence of exposed core part results into the dumbbell-like core/shell-core-core/shell triblock nanowires with the distinct height-graded at the junction along the axis. Therefore, the sequential self-assembly approach based on the manipulation of the hierarchical noncovalent interaction is of both fundamental and technological significance to the development of precisely constructing OHSs, as well as integrated optoelectronics such as optical logic gate operation with multiple input/out channels and high-fidelity signal.

## Results

### Strategies for hierarchical self-assembly of 1D OHSs

**Fig. 1:**
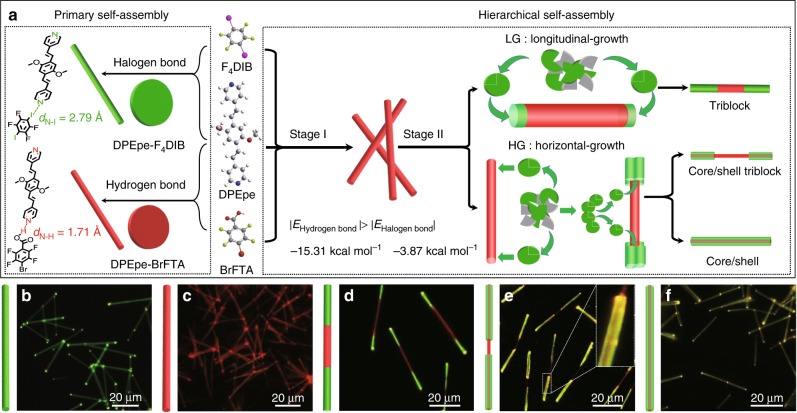
Illustration of the hierarchical self-assembly for 1D OHSs. **a** Schematic illustrations: the primary self-assembly of halogen-bonded DPEpe-F_4_DIB cocrystal and hydrogen-bonded DPEpe-BrFTA cocrystal; the hierarchical self-assembly of organic TNWs, DB-C/S-NWs, and organic C/S-NWs. **b**–**f** FM images of these as-prepared **b** halogen-bonded DPEpe-F_4_DIB cocrystals, **c** hydrogen-bonded DPEpe-BrFTA cocrystals, **d** organic TNWs, **e** organic DB-C/S-NWs and **f** organic C/S-NWs excited with the UV band (*λ* = 330–380 nm) from a mercury lamp. Scale bars are all 20 μm. Inset of (**e**): the magnified image of a typical tip in DB-C/S-NWs, clearly showing the core-shell structure

The multifunctional pyridine group was widely applied to form organic cocrystals based on the hydrogen/halogen bonds^38–41^, and the charge-transfer interaction^42,43^. From the primary self-assembly process (Fig. 1a), the 4,4’-((1*E*,1’*E*)-(2,5-dimethoxy-1,4-phenylene)bis(ethene-2,1-diyl))dipyridine (DPEpe) and 1,4-diiodotetrafluorobenzene (F_4_DIB) self-assemble into DPEpe-F_4_DIB nanowires via the halogen bond. The halogen-bonded DPEpe-F_4_DIB nanowires display the green emission under the excitation of UV-band (330–380 nm) (Fig. 1b). In the Infrared (IR) spectra (Supplementary Fig. 1), the bands at 758 cm^−1^ (C-I antisym str) and 1465 cm^−1^ (aryl semicircle stretch) of F_4_DIB respectively move to 744 cm^−1^ and 1446 cm^−1^ of DPEpe-F_4_DIB, which is attributed to the *n* *→* σ* donation and the N···I halogen bond formation in DPEpe-F_4_DIB^43^. Likewise, DPEpe and 4-bromo-2,3,5,6-tetrafluorobenzoic acid (BrFTA) can self-assemble into the hydrogen-bonded DPEpe-BrFTA nanowires with red emission under the excitation of UV-band (Fig. 1c). The 1706 cm^−1^ band (C = O str) and 1476 cm^−1^ band (aryl semicircle stretch) of the BrFTA become weak after co-crystallization, which verifies the formation of the hydrogen bond (N···H-O) in the DPEpe-BrFTA (Supplementary Fig. 1)^44^. The normalized contacts distance of halogen bond (*R*_N···I_ = 0.79) for DPEpe-F_4_DIB cocrystal is longer than that of hydrogen bond (*R*_N-H_ = 0.63) for DPEpe-BrFTA cocrystal^45^. Combining with DFT calculation for the noncovalent interaction strength: |*E*_hydrogen bond_ = −15.66 kcal mol^−1^| > |*E*_halogen bond_ = −4.90 kcal mol^−1^| > |*E*_π–π interaction_ = −0.09 kcal mol^−1^| (Supplementary Fig. 2), the hydrogen bond leads to the first order self-assembly process for DPEpe-BrFTA cocrystals as preformed seed nanowires at stage I in the hierarchical self-assembly processes (Fig. 1a). Then, the DPEpe-F_4_DIB cocrystal epitaxial growth on tips or side surfaces of the DPEpe-BrFTA nanowires to obtain the LG of triblock heterostructure or HG of core/shell heterostructure in the stage II by controlling the temperature of the stock solution. With certain halogen bond acceptor F_4_DIB only for the partial HG, the core part at center of the nanowires will be exposed and the dumbbell-like core/shell nanowires (DB-C/S-NWs) will be obtained. Well-defined green-red-green TNWs and dumbbell-like DB-C/S-NWs were preliminarily confirmed by the FM images in Figs. 1d, e, respectively. As illustrated in the inset of Fig. 1e, the nanowire displays the bright yellow-emitting shell with a red-emitting wire in the core, thus suggesting the formation of the core/shell structure in DB-C/S-NWs. Likewise, the whole C/S-NWs with yellow emission were indicated in Fig. 1f. Furthermore, the IR spectra of the heterostructure interfaces in both the triblock and the core/shell heterostructure nanowires (Supplementary Fig. 1) have the characteristic IR bands of the halogen bond at 744, 928, 1590, and 1625 cm^−1^ and the hydrogen bond at 738, 965 1415, and 1706 cm^−1^, which verifies the formation of the halogen and hydrogen bonds in the interface. Based on the aforementioned sequential crystallization process, the OHSs with DPEpe-BrFTA nanowires that located in the center part of the triblock structure or in the core part of the core/shell structure are constructed.

### LG self-assembly for triblock heterostructure nanowires

**Fig. 2:**
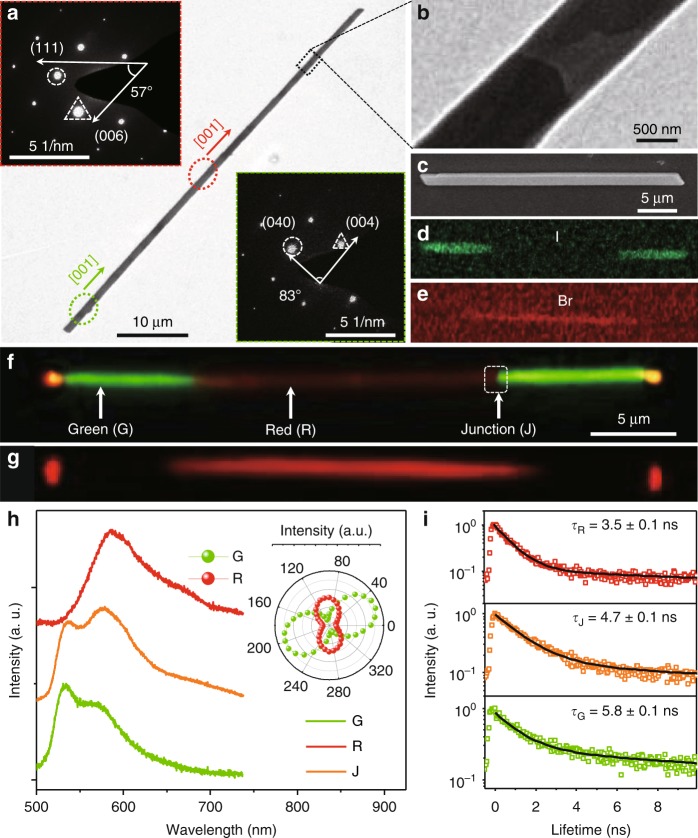
Structure and optical characteristics of TNWs. **a** The TEM image of one typical TNW, scale bar: 10 μm. The SAED patterns at the upper-left and the below-right insets correspond to the center part with red marked area and the end part green marked area in TNWs, respectively. **b** The magnified image of the junction in the TNW, scale bar: 500 nm. **c** The SEM image of one typical TNW; scale bar: 5 μm. The corresponding energy-dispersive X-ray spectroscopy (EDS) mapping for elements (**d**) iodine (I) and (**e**) bromide (Br), respectively. The FM images of one typical TNW excited with (**f**) the UV band and (**g**) the green band (500–550 nm), scale bar: 5 μm. **h** Spatially resolved PL spectra and **i** PL decay curves collected from different locations marked in (**f**). Inset of **h**: the corresponding polar image of the peak intensities

Through a LG hierarchical self-assembly process, the organic triblock herterostructure (TNWs) were successfully prepared with the stock solution at 25 °C. The selected area electron diffraction (SAED) patterns of the end (center) part (the insets of Fig. 2a) exhibit the identical pattern as that of DPEpe-F_4_DIB (DPEpe-BrFTA) cocrystal (Supplementary Fig. 2). Combining the X-ray diffraction (XRD) pattern of the TNWs (Supplementary Fig. 4) including the characteristic diffractions of both DPEpe-BrFTA and DPEpe-F_4_DIB cocrystal, it indicates that the end and center parts in the TNWs respectively correspond to DPEpe-F_4_DIB and DPEpe-BrFTA cocrystal with the high crystallinity and the same growth direction of [001] (Fig. 2a). Moreover, the high-resolution transmission electron microscopy (TEM) image with the obvious contrast (Fig. 2b) and the SAED pattern with two sets of diffraction spots (Supplementary Fig. 3c) indicates the existence of the junction. As shown in Fig. 2c–e and Supplementary Fig. 5a the scanning electron microscopy (SEM) energy-dispersive X-ray spectroscopy (EDS) mappings on a TNW show a clear interface between the bromide section (red at center) and iodine section (green at two end), whereas the carbon is uniformly distributed within the whole nanowire (Supplementary Fig. 6). Thus, these above-mentioned results imply that the end and center parts in the TNWs are made up of DPEpe-F_4_DIB and DPEpe-BrFTA cocrystal, respectively.

The fluorescence microscopy (FM) images of the TNWs (Fig. 2f, g) demonstrate that center part emits red light under the excitation of both the UV band and the green band (500–550 nm), while the end parts can be only excited by the UV band. This excitation wavelength-dependent emission is attributed to the spectral overlap between the excitation source and the intrinsic excitation spectra of DPEpe-BrFTA and DPEpe-F_4_DIB cocrystal (Supplementary Fig. 7b). As shown in the inset of Fig. 2h, the polarized angles *θ* for the highest PL intensities of the PL emitted from the end part (525 nm) and center part (600 nm) are 20° and 90°, which further confirms the different crystal structures in the TNWs. The PL emission characteristics and the corresponding PL lifetime of center (end) part (Fig. 2h, i) are in good consistence with that of DPEpe-BrFTA (DPEpe-F_4_DIB) cocrystal (Supplementary Fig. 7a). Besides, the spectrum of the junction includes the green emission from DPEpe-BrFTA cocrystal and red emission from DPEpe-F_4_DIB cocrystal, and the corresponding PL lifetime decreases from 5.8 ns of DPEpe-F_4_DIB cocrystal to 4.7 ns, owing to the energy transfer process from DPEpe-F_4_DIB to DPEpe-BrFTA cocrystal. Furthermore, the organic triblock heterostructure nanowires prepared before 90 days demonstrate the same morphology and the remained optical property as those of the fresh samples (Supplementary Fig. 8a-c), which indicates the stability of these triblock heterostructure nanowires. Thus, we have synthesized the organic triblock heterostructure comprised the DPEpe-F_4_DIB cocrystal at the end and the DPEpe-BrFTA cocrystal at center via a LG hierarchical self-assembly process.

### Elaborate control for triblock heterostructure nanowires

**Fig. 3:**
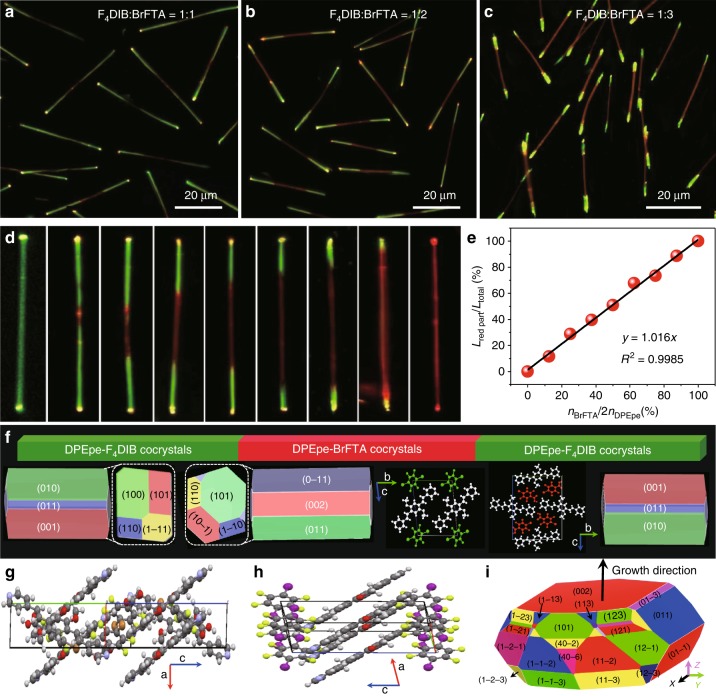
Elaborate control for the structure and growth mechanism of the TNWs. **a–c** FM images of TNWs excited with the UV band based on the different molar ratios between F_4_DIB and BrFTA: **a** 1:1, **b** 1:2 and **c** 1:3, scale bars are all 20 μm. **d** FM images of the TNWs with increasing molar ratio of BrFTA from left to right. **e** Plot of the length ratio of red region in total wire versus the molar ratios between the hydrogen acceptor of BrFTA and the hydrogen/halogen donors of DPEpe. **f** BFDH morphologies, the molecular packing arrangements in *bc* plane and end crystal faces both in the DPEpe-BrFTA and DPEpe-F_4_DIB cocrystal. **g** The molecular packing arrangements of the *ac* plane of **g** DPEpe-BrFTA cocrystal and **h** DPEpe-F_4_DIB cocrystal. **i** The simulated equilibrium morphology of DEPpe-BrFTA cocrystals. The simulated BFDH and equilibrium morphologies base on the interplanar spacing and the minimum total surface energy using the Materials Studio software package, respectively

Notably, the length of the DPEpe-F_4_DIB or DPEpe-BrFTA cocrystal in the TWNs can be quantitatively controlled via adjusting the formation of hydrogen/halogen bonds in the LG hierarchical self-assembly process. As indicated in Fig. 3a–d and Supplementary Fig. 9, the length ratio of the red-emissive DPEpe-BrFTA cocrystals under the excitation of UV-band in the TNWs is proportional to the ratio of hydrogen-bonded acceptor BrFTA. Remarkably, it is found that the length ratio of the DPEpe-BrFTA cocrystals at center in the triblock nanowires is linear-dependent on the ratio between the BrFTA and the DPEpe as verified in Fig. 3e. Therefore, the accurate quantitative operation of the desired axial striped structure in TNWs is rationally controlled through adjusting the ratio of halogen bond acceptor F_4_DIB and hydrogen bond acceptor BrFTA based on the linear-dependent relationship.

The molecular packing arrangements in DPEpe-F_4_DIB and DPEpe-BrFTA cocrystal is worth to evaluate the mechanism of LG hierarchical self-assembly process. As shown in Fig. 3f, the small lattice mismatch of the (001) surface and the same 1D rod-like Bravais-Friedel-Donnay-Harker (BFDH) morphologies preferentially favor the anisotropic growth of DPEpe-F_4_DIB cocrystals onto the tips of the DPEpe-BrFTA cocrystal along the *c*-axis. The similar lattice constants of the crystal faces at the end in the two BFDH morphologies (Supplementary Table 3: *d*_{111}s_ = 3.71 Å, *d*_{101}s_ = 3.80 Å, *d*_{10-1}s_ = 3.86 Å, *d*_{110}s_ = 3.78 Å for DPEpe-BrFTA and *d*_{111}s_ = 3.99 Å, *d*_{101}s_ = 4.04 Å, *d*_{100}s_ = 4.06 Å, *d*_{110}s_ = 3.96 Å for DPEpe-F_4_DIB) facilitate the LG along the *c* axis. Moreover, the DPEpe molecules have the same packing arrangement on the *bc* plane with an angle of 37^o^ along *c*-axis in the two crystal structures (Fig. 3g, h), which leads to the facile replacement between the F_4_DIB and BrFTA, inducing the selective nucleation and the epitaxial growth of DPEpe-F_4_DIB. Consequently, the lattice matching in both the *c* axis and the interplanar-spacing of crystal face at the end in the BFDH morphologies preferentially favors the LG for the realization of the 1D organic TNWs under the kinetically equilibrium with stock solution at 25 °C. As is known to us, the stock solution temperature is important for the controllable construction of nanomaterials with desired morphology under kinetically or thermodynamic equilibrium^46,47^. The surface energy of the lateral-crystal-faces (Supplementary Table 4: *E*_*surf {011}s*_ (26.2 kcal mol^−1^) ≤ *E*_surf lateral-crystal-faces_ ≤ *E*_*surf {40-2}s*_ (161.9 kcal mol^−1^)) in the equilibrium morphology of DPEpe-BrFTA cocrystal for thermodynamic equilibrium (Fig. 3i) is as least as four-folds larger than that (8.1 kcal mol^−1^) of the top/bottom facets (002)s. The surface-interface energy balance also prefers DPEpe-F_4_DIB nucleation at the lateral facets to eliminate the unstable or high energy facets in DPEpe-BrFTA cocrystal, leading to the HG and thermodynamically favorable state, as in the case of *b*-TiO_2_-Fe_x_O_y_ nanorods heterostructures^48^ and coronene-perylene side surface growth heterostructures^17^. As a result, the surface-interface energy balance facilitates the HG of DPEpe-F_4_DIB on the side surfaces of the preformed DPEpe-BrFTA, which results into a core/shell structure under the thermodynamical equilibrium with a stock solution at high temperature.

### HG self-assembly for core/shell heterostructure nanowires

**Fig. 4:**
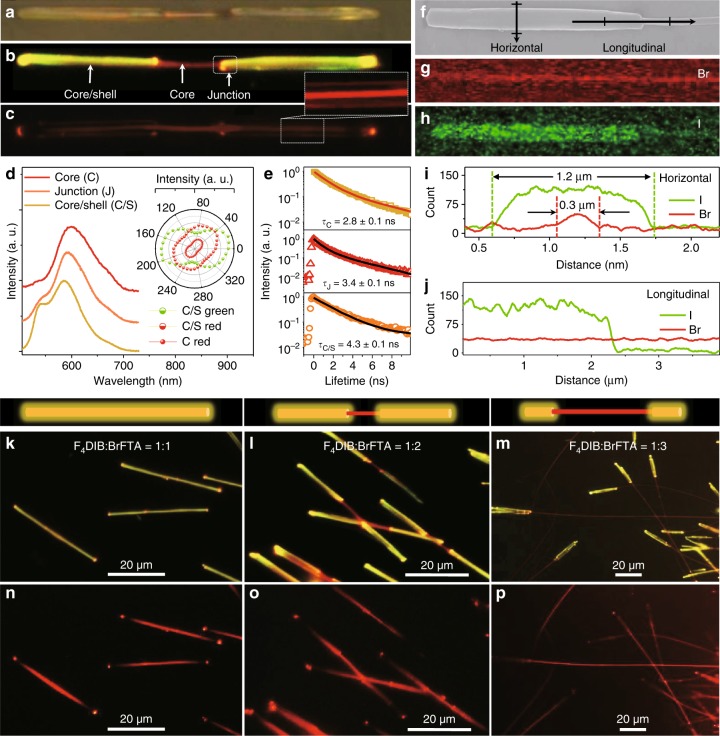
Structure and optical characteristic of C/S-NWs. **a** Optical image and corresponding FM images under excitation by **b** the UV band and **c** green-light of an individual DB-C/S-NW with scale bar of 5 μm. **d** Spatially resolved PL spectra and **e** PL decay curves collected from different locations marked in (**b**). Inset of **d**: corresponding polar image of the peak intensities. **f** The SEM image of a typical DB-C/S-NW and the corresponding EDS mapping for **g** Br and **h** I; scale bar: 5 μm. The element I and Br distribution along **i** vertically and **j** parallel directions marked in d_1_. FM images of DB-C/S-NWs based on different ratio between the halogen bond acceptor of F_4_DIB and the hydrogen bond acceptor of BrFTA: **k**, **n** 1:1, **l**, **o** 1:2 and **m**, **p** 1:3; scale bar: 20 μm. The excitation source for (**k–m**) and (**n–p**) are the UV light and green-light, respectively

According to the aforementioned discussed mechanism, the FM images of the organic 1D heterostructures (Supplementary Fig. 12) show that the triblock structure is hard to recognize with the stock solution at temperature from 25 to 40 °C, as well as the core/shell structure evolves more obviously with the stock solution at temperature from 40 to 55 °C. Besides, the unordered structure nanowires with random emission color (Supplementary Fig. 12d, e) have been prepared with the stock solution at 40 °C, as well as DB-C/S-NWs with recognizable yellow-red-yellow color-display under the UV-band excitation (Fig. 4b) have been prepared with the stock solution at 55 °C. The bright red-emission at center and the yellow-emission at ends under UV irradiation (Fig. 4b), as well as the bright red-emission at the whole axes in the bulky rod-like end under green-band excitation (Fig. 4c), clearly show the core/shell structure at the end and the exposed core at center. As illustrated in Fig. 4d, the spatially resolved PL spectra collected from different locations marked in Fig. 4b shows that the exposed core has the consistent red-emission with of DPEpe-BrFTA cocrystal (Supplementary Fig. 7). In contrast, an additional green light emitted from the shell of DPEpe-F_4_DIB cocrystal appears on the PL spectrum of the end part. Due to the energy transfer from the shell of DPEpe-F_4_DIB cocrystals to core of DPEpe-BrFTA cocrystals, the PL lifetime corresponding to the emission at the end is 4.3 ns (Fig. 4e), which is longer than that of the DPEpe-BrFTA cocrystals (2.9 ns) and shorter than that of the DPEpe-F_4_DIB cocrystals (4.5 ns). Due to the core/shell structure, the anisotropy polarizations of PL emission from end part demonstrate an angle of 30° at peak between 525 nm and 600 nm (the inset of Fig. 4d). The isotropy polarizations of red-emission (600 nm) from the end and center part indicate the same crystal structure of the whole core in the DB-C/S-NWs. As shown in Fig. 4f–h and Supplementary Fig. 5b about the SEM EDS mappings of the core/shell nanowires, the bromide section (red at center along the whole axes) and iodine section (green just appeared at the end) are corresponding to the core part of DPEpe-BrFTA cocrystal and the shell part of DPEpe-F_4_DIB cocrystal, which is further affirmed by the TEM image with SAED patterns (Supplementary Fig. 13). The distributions of bromide and iodine (Fig. 4i) along the horizontal direction at the end (marked in the Fig. 4h) are proportional to the height profile of the end part and center part, respectively. By contrast, the bromide (iodine) along the longitudinal axes of the TNWs marked in Fig. 4h distribute whole axes (the only end part). Thus, the thermodynamically favorable state of core/shell heterostructure nanowires (including DB-C/S-NWs) comprised the DPEpe-F_4_DIB cocrystal at the shell and the DPEpe-BrFTA cocrystal at core, which prepared via the HG under the stock solution temperature at 55 °C.

Impressively, the exposed degree of the core at center part in the C/S-NWs can be quantitatively controlled via tuning the formation ratio of hydrogen/halogen bonds in the HG hierarchical self-assembly process under the stock solution temperature at 55 °C. With a high ratio of F_4_DIB, the DPEpe-F_4_DIB cocrystal can thoroughly epitaxially grow on the side of the preformed DPEpe-BrFTA nanowires, leading to the whole DPEpe-BrFTA/DPEpe-F_4_DIB C/S-NWs with bright yellow emission under the UV-band excitation, as verified by the FM image (Fig. 4k) and the confocal laser scanning microscopy image (Supplementary Fig. 14). With a low ratio of F_4_DIB in contrast, the core at the center will not be coated by the DPEpe-F_4_DIB cocrystal, resulting into the DB-C/S-NWs as demonstrated by FM image in the Fig. 4l. As shown in Fig. 4m and Supplementary Fig. 15, the exposed degree of the core at center in DB-C/S-NWs increases after further decreasing the ratio of F_4_DIB. Under the green-band excitation, just the core of DPEpe-BrFTA cocrystals displays the red-emission in both the whole C/S-NWs and the DB-C/S-NWs as shown in Fig. 4n–p. Additionally, the organic core/shell heterostructure nanowires prepared before 90 days have the same dumbbell-like 1D structure and the remained optical property with the fresh samples (Supplementary Fig. 8d–f), which proves the stability of these organic core/shell heterostructure nanowires.

### Optical logic gate operation of OHS nanowires

The photon propagation in the single-component organic micro-/nanostructures is the necessary to evaluate photon propagation at the heterojunction, which is considered for their potential applications, such as multi-color optical waveguide or optical logic gate^49^. The micro-area FM images and the distance-dependent emission spectra (Supplementary Fig. 16) were performed by accurately shifting the 375 nm excitation laser spots along the organic nanowires. Then, the corresponding optical-loss coefficients (*R*_DPEpe-BrFTA_ = 0.023 dB μm^−1^ and *R*_DPEpe-F4DIB_ = 0.030 dB μm^−1^) were calculated out based on the single-exponential decay of the *I*_tip_/*I*_body_ against the propagation distance^46^. Based on the difference of bandgap (Δ*E*_g_) between the excited and emitted position media, the photon propagation at the heterojunction demonstrates the active waveguide (Δ*E*_g_ ≥ 0) and passive waveguide (Δ*E*_g_ < 0)^24,25^. The spectrum and corresponding polarizations of the emitted photons for the active (passive) waveguide are in accord with that of emitted (excited) position media (Supplementary Figure 17). Based on the TNWs (Supplementary Fig. 18a–c), the emission color of the emitting tip changes from green to red during shifting the excitation laser spots (*λ* = 375 nm) from left to right and orderly undergoing the active and the passive waveguide with *R*_active_ = 0.037 dB μm^−1^ and *R*_passive_ = 0.043 dB μm^−1^. The DB-C/S-NWs also demonstrate the multi-color optical waveguide with four output channels (Supplementary Figure 18d, e).

**Fig. 5:**
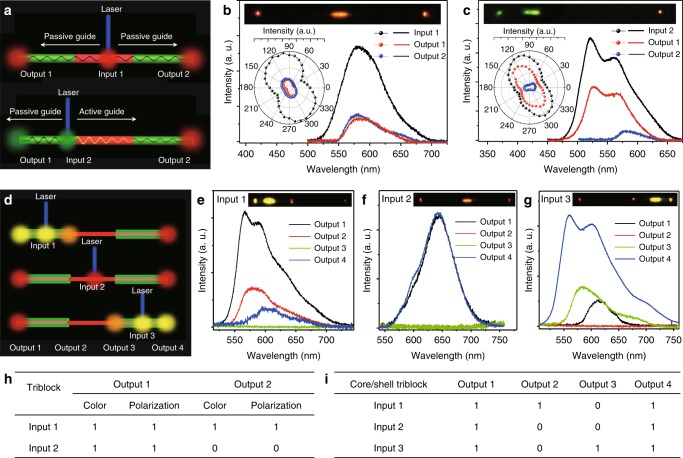
Optical logic gate operation of 1D OHSs. **a** Schematic diagram of optical logic gate based on the organic TNW. **c**, **d** Spatially resolved PL spectra collected from two tips after exciting with a laser beam (*λ* *=* 375 nm) at the center part (**b**) and the end part (**c**). Inset: the corresponding FM image and polar image of the peak intensities. **d** Schematic diagram of optical logic gate based on the organic DB-C/S-NWs. **e**–**g** Spatially resolved PL spectra of the four output channels corresponding three input channels as marked in the (**d**). Inset: the corresponding FM images. **h**, **i** The summarized optical logic operation information code for (**h**) organic TNW and (**i**) organic DB-C/S-NWs

The heterostructure waveguides with the spatially dependent multicolor emission exhibit a variety of potential applications in organic photonic circuits, such as the wavelength converter^26^, photonic transistor^27^, and optical router^28^. Therefore, the excitation position-dependent multicolor emission properties with the selective wavelength and polarization of the TNWs (Fig. 5a) and selective output emission of the DB-C/S-NW (Fig. 5d) were applied in the optical logic gate operation and encoding/decoding with multiple input/output channels at nanoscale. When excited at center part in the TNW (Fig. 5b), the photon propagation to the two ends is passive waveguide, leading to identical emission color and polarization at the tips with that of the excited position. In contrast with the excited position at the end of the TNW (Fig. 5c), the photon propagation to the left-end (right-end) with short (long) transmission distance corresponds to the passive (active) waveguide, leading to the identical emission color and polarization at the tips with that of the excited position (right-end position). The active and passive waveguide respectively correspond to logic gate signal of 0 and 1. Due to the large angle shift of ~70° in the polarization and the huge wavelength shift of ~60 nm in the emission spectra (Fig. 5c), the signal of 0 and 1 can be absolutely recognized, which leads to high-fidelity signal. The truth table of these logic gate functions is summarized in Fig. 5h. For the DB-C/S-NW, there are four output terminals located on two tips and two junctions as shown in the Fig. 5d. When excited at Input 1, except the Output 3, other output terminals are bright (Fig. 5e). When excited at Input 2, the Output 1 and Output 4 are bright, and the Output 2 and Output 3 are dark (Fig. 5f). When excited at Input 3, except the Output 2, other output terminals are bright (Fig. 5g). The bright and dark state of the output terminals correspond to logic gate signal of 0 and 1, respectively. The optical logic gate based on the DB-C/S-NW also demonstrated the high-fidelity signal owing to the absolute dark-state. The truth table of these logic gate functions is summarized in the Fig. 5i. Due to the triblock heterojunction and the core/shell structure with anisotropy optical character for photons propagation, the prepared heterostructure nanowires realize the optical logic gate with high-fidelity signal.

## Discussion

In summary, the organic triblock and the core/shell (including DB-C/S-NWs) heterostructure nanowires were successfully prepared via a hierarchical self-assembly based on the precise manipulation of noncovalent interactions: hydrogen and halogen bonds, π–π interaction. The kinetically controlled realization of TNWs comprising DPEpe-F_4_DIB cocrystal at end parts and DPEpe-BrFTA cocrystal at center part is attributed to the small lattice mismatch under the stock solution at 25 °C. Likewise, the thermodynamically controlled realization of DB-C/S-NWs comprising DPEpe-F_4_DIB cocrystal as shell and DPEpe-BrFTA cocrystal as core is attributed to the surface-interface energy balance under the stock solution at 55 °C. Owing to the triblock and core/shell structure with anisotropy optical character, these as-prepared OHSs nanowires demonstrate the excitation position-dependent optical waveguide with the polarization-angle/wavelength-shift-dependent emission and the absolute dark state, which function as the nanoscale multiple in/output optical logic gate with absolute fidelity signal. The present strategy develop a method based on manipulating the diverse weak noncovalent interactions for the precisely constructing organic hierarchical micro-nanostructure with both fundamental and technological significance, which will guide the materials chemistry upsurge on the OHSs.

## Methods

### Materials

1,4-Diiodotetrafluorobenzene (F_4_DIB, 98%) and 4-bromo-2,3,5,6-tetrafluorobenzoic acid (BrFTA, 97%) were purchased from Sigma-Aldrich Co.. 2,5-bismethoxy-1,4-xylene-bis(diethyl phosphonate), isonicotinaldehyde, NaH, 2,4,5-tetrafluoro-3,6-diiodobenzene (98%) was purchased from Sigma-Aldrich. The chloroform (CHCl_3_, analysis grade), tetrahydrofuran (THF, HPLC grade), hexane and ethyl alcohol (analysis grade) solvents were purchased from Beijing Chemical Agent Ltd., China. In addition, all compounds and solvents were used without further treatment. Ultrapure water with a resistance of 18.2 MΩ cm^−1^, produced by using a Milli-Q apparatus (Millipore), were used in all experiments. Alumina membranes with a pore size of 20 nm and polytetrafluoroethylene filters (PTFE, Puradisc 25 TF, 0.1 μm) were bought from Whatman International Ltd.

### Organic crystal growth

The individual DPEpe-F_4_DIB or DPEpe-BrFTA cocrystals were obtained via the primary self-assembly process by the slow evaporation of the solvents. In a typical experiment, 0.1 mmol DPEpe and 0.1 mmol F_4_DIB (0.2 mmol BrFTA) were dissolved in mixed solvent including 10 mL chloroform and 40 mL ethyl alcohol in a 100 mL vial at room temperature. The concentrations of the stock solution corresponding to DPEpe, F_4_DIB and BrFTA are 10.0, 10.0, and 20.0 mmol L^−1^, respectively. Then, the dispersed solution was dropped onto a quartz substrate, and cocrystals were observed after the solvent was evaporated completely. The 1D organic heterostructure nanowires were obtained via the longitudinal/horizontal hierarchical self-assembly process by the slow evaporation of the solvents. In a typical experiment for the realization of longitudinal growth green-red-green triblock nanowires, 0.1 mmol DPEpe, *x* mmol F_4_DIB and y mmol BrFTA (*x* + 0.5 *y* = 0.1) were dissolved in mixed solvent including 10 mL chloroform and 40 mL ethyl alcohol in a 100 mL vial at room temperature (25 °C). Then, the dispersed solution was dropped onto a quartz substrate, and heterostructure nanowires were observed after the solvent was evaporated completely. In a typical experiment for the realization of horizontal growth core/shell nanowires, 0.1 mmol DPEpe, *x* mmol F_4_DIB and y mmol BrFTA (*x* + 0.5 *y* = 0.1) were dissolved in mixed solvent including 10 mL chloroform and 40 mL ethyl alcohol in a 100 mL vial at room temperature. Then, the dispersed solution was put in the water bath (55 °C) keeping for 30 min and dropped onto a quartz substrate, and heterostructure nanowires were observed after the solvent was evaporated completely.

### Micro-area photoluminescence spectra measurement

Micro-area photoluminescence (*μ*-PL) spectra and PL decay curves were collected on a homemade optical microscopy (Supplementary Fig. 19). To measure the PL spectra of individual microplate, the micro/nanostructure was excited locally with a 375 nm laser focused down to the diffraction limit. The excitation laser was filtered with a 375 nm notch filter. The light was subsequently coupled to a grating spectrometer (Princeton Instrument, ARC-SP-2356) and recorded by a thermal-electrically cooled CCD (Princeton Instruments, PIX-256E). PL microscopy images were taken with an inverted microscope (Olympus, BX43). The spatial-based decay lifetime was achieved via a spatially/temporally/spectrally-resolved confocal imaging system of ISS Q2 Confocal laser scanning nanoscope (Detection Spectral Range: 350–1050 nm, Lifetime Measurement Range: 100 ps-100 ms, Optical Resolution: diffraction limited down to 250 nm). The polar image of the peak intensities was collected via tuning the angle of polarization analyzer on the homemade optical microscopy.

## Supplementary information

Supplementary Information

## Data Availability

All data needed to evaluate the conclusions in the paper are present in the paper and/or the Supplementary Materials. Additional data related to this paper may be requested from the authors.
