# Supplementary material for: Hierarchical self-assembly of organic heterostructure nanowires
Source: Nat Commun. 2019 Aug 26;10:3839. doi: 10.1038/s41467-019-11731-7 (PMC6710268; doi:10.1038/s41467-019-11731-7)
Supplement: Supplementary file 1 — Supplementary Information [file 41467_2019_11731_MOESM1_ESM.pdf]

Supporting Information

Hierarchical Self-assembly of Organic Heterostructure Nanowires

Ming-Peng Zhuo[1], Jun-Jie Wu[1], Xue-Dong Wang[1]*, Yi-Chen Tao[1], Yi Yuan[1], Liang-Sheng Liao[1,2]*

[1]Institute of Functional Nano & Soft Materials (FUNSOM), Jiangsu Key Laboratory for Carbon-Based Functional Materials & Devices, Soochow University, 199 Ren'ai Road, Suzhou, Jiangsu 215123, P. R. China.

[2]Institute of Organic Optoelectronics, JITRI, Wujiang, Suzhou, Jiangsu 215211, P. R. China.

*E-mail: wangxuedong@suda.edu.cn (X.-D. Wang); lsliao@suda.edu.cn (L.-S. Liao)

Supplementary Methods

1. **Synthesis procedure of 4,4'-((1*E*,1'*E*)-(2,5-dimethoxy-1,4-phenylene)bis(ethene-2,1-diyl))dipyridine (DPEpe)**

   A mixture of 2,5-bismethoxy-1,4-xylene-bis(diethyl phosphonate) (1.01 g, 2.26 mmol) and the isonicotinaldehyde (0.48 g, 4.52 mmol) in THF cooled in an ice bath was added 2 *eq*. NaH in small portions during a 30 min period. The reaction mixture was stirred at room temperature for 3 h and poured into water. The phase was extracted with CH_2Cl_2. The pooled organic phases were washed with water, dried over anhydrous $MgSO_4$, filtered, and evaporated. The product was separated by flash chromatography on silica gel by means of CH_2Cl_2/petroleum ether (1:4). Finally a highly fluorescent yellow powder was obtained as the title compound (0.655 g, 1.82 mmol) in 81% yield. ^{1}H NMR (400 MHz, Chloroform-*d*) δ 8.55 (*d*, J = 5.4 Hz, 4H), 7.70 (*s*, 1H), 7.66 (*s*, 1H), 7.54 (*d,* J = 5.5 Hz, 4H), 7.41 (*s*, 3H), 7.36 (*s*, 1H), 3.92 (*s*, 6H).

2. **Organic crystal characterizations**

The morphology, size and typical element distribution of the organic micro/nanostructures were examined by field emission scanning electron microscopy (FESEM, Carl Zeiss, Supra 55, Germany) with a 20 *X*-MaxN Energy Dispersive Spectrometer (EDS, Oxford Instruments, United Kingdom) dropping on an indium tin oxide (ITO) coated glass. The elemental measurement was performed by energy-dispersive *X*-ray (EDX) mapping mode. TEM images were obtained by a transmission electron microscopy (TEM, FEI company, Tecnai G2 F20, United States). One drop of the solution was dropped on a carbon-coated copper grid, and evaporated. TEM measurement was performed at room temperature at an accelerating voltage of 100 kV. The *X*-ray diffraction (XRD) patterns were measured by a D/max 2400 *X*-ray diffractometer with Cu Kα radiation (λ = 1.54050 Å) operated in the 2θ range from 5° to 30°, by using the samples on the quartz. Fluorescence images were recorded using a fluorescence optical microscope (Leica, DM4000M, Germany) with a spot-enhanced charge couple device (Diagnostic Instrument, Inc.). The excitation source is a mercury

lamp equipped with a band-pass filter (330-380 nm for UV-light and 500~550 nm for green-light). The samples were prepared by placing a drop of solution onto a cleaned quartz, and then evaporated at room temperature.

Supplementary Figures

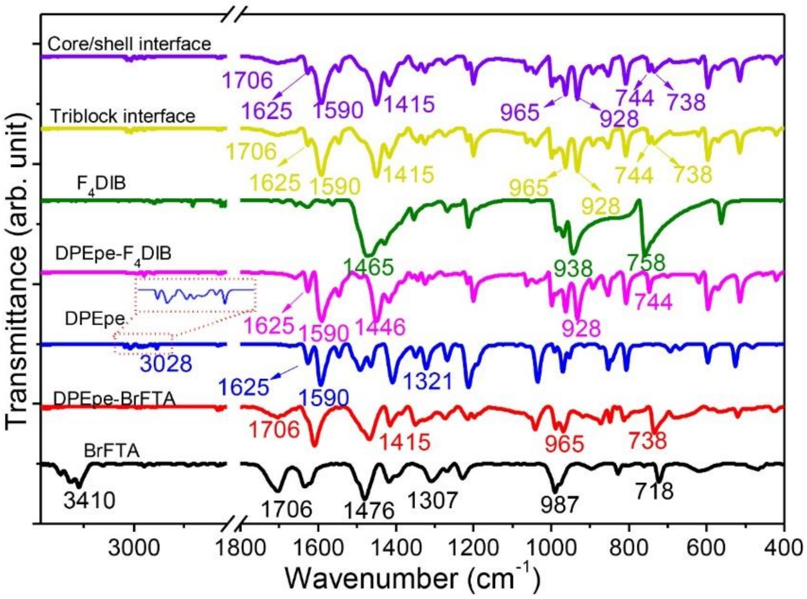

Supplementary Figure 1. Infrared (IR) spectra of BrFTA (black line), DPEpe (blue line) and F4DIB (green line) powder, DPEpe-BrFTA nanowire (red line), DPEpe-F4DIB nanowire (pink line), interface in both triblock (yellow line) and core/shell (purple line) heterostructure nanowire.

The IR spectra of the powders and the organic micro-/nanocrystals were obtained via the traditional pressed-disk technique and the micro-area technique (Bruker, HYPERION), respectively. As shown in the Supplementary Figure 1, the 758 cm^{-1} of the F_4DIB (C-I antisym str)[1] moves to 744 cm^{-1}, and the 1465 cm^{-1} (aryl semicircle stretch)[2] changes to 1446 cm^{-1} after co-crystallization. All of these signals indicate that the C-I bond is lengthened and weakened as a consequence of $n \rightarrow \sigma^*$ donation and N···I halogen bond formation in DPEpe-F_4DIB.[3] Furthermore, the shift of the 938 cm^{-1} of the F_4DIB (C-F antisym str) to 928 cm^{-1}, corresponds to the C-F···H interaction in DPEpe-F_4DIB. Additionally, the largely weakened intensity of both the 3028 cm^{-1} (C-H str) and 1321 cm^{-1} (C−N str) in DPEpe after co-crystallization further confirms that the halogen bond exists in the DPEpe-F_4DIB.[3] Besides, the 718 cm^{-1} peak (C-Br antisym str) of the BrFTA moves to 738 cm^{-1}, which corresponds to formation of the C−Br···O=C in the DPEpe-BrFTA. Under the effect of a strong pyridine ring mode, the 1307 cm^{-1} peak of the γ-OH moves to 1415 cm^{-1}, as well as the 987 cm^{-1} peak (C-F

antisym str) moves to 965 cm^{-1} with the C-F···H interaction in DPEpe-BrFTA.[4] Due to the proton-stretching motion with a low barrier separating the two minima, the disappeared *v*-OH at 3410 cm^{-1} corresponds to a single minimum potential function for the hydrogen bond between the pyridine and the acid.[5,6] The 1706 cm^{-1} IR band (C=O str) and 1476 cm^{-1} (aryl semicircle str) in BrFTA after co-crystallization become weak, which verifies the formation of the hydrogen bond (N···H-O) in the DPEpe-BrFTA.[7] Furthermore, the largely weakened intensity of both the 3028 cm^{-1} (C-H str) and 1321 cm^{-1} peak (C-N str) in DPEpe after co-crystallization further confirms the formation of hydrogen bond in the DPEpe-BrFTA.

Compared with both the halogen-bonded DPEpe-F_4DIB and the hydrogen-bonded DPEpe-BrFTA, the IR spectra of the heterostructure interface (Supplementary Figure 1) in the triblock (deep blue line) and core/shell (purple line) heterostructure nanowire have the IR band at 744, 928, 1590, and 1625 cm^{-1}, which verifies the formation of the halogen bond. Furthermore, the IR band at 738, 965, 1415, and 1706 cm^{-1} verifies the formation of the hydrogen bond.

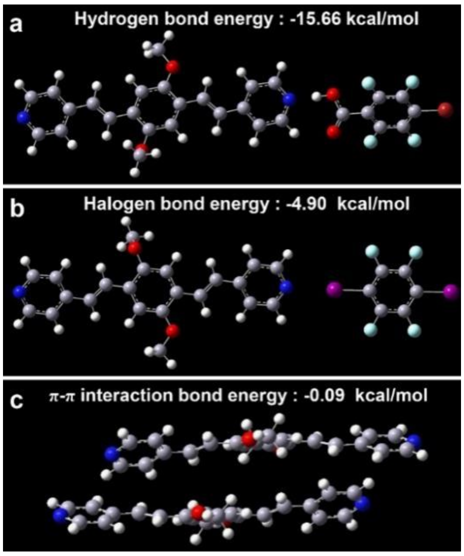

Supplementary Figure 2. Optimized molecular structure of (a) hydrogen bond DPEpe-BrFTA, (b) halogen bond DPEpe-F_4DIB and (c) π-π interaction of DPEpe-DPEpe. The TD-DFT calculations for the bond energy: $E_{\text{hydrogen bond}}$ = -15.66 kcal mol^{-1}, $E_{\text{halogen bond}}$ = -4.90 kcal mol^{-1} and $E_{\pi\text{-}\pi\text{ interaction}}$ = -0.09 kcal mol^{-1}. The interaction energy between organic molecules were performed by using Gaussian 09 software package[8] with 6-31+*G*(*d, p*) basis set and B3lyp level of theory[9] after structure optimization in the vacuum, which was conducted according to the following equation considering the Basis Set Superposition Error (BSSE) correction: $E_{int} = E_{tot} - (E_1 + E_2) + E_{bsse}$, E_{int} is the interaction energy between the two molecules, E_1 and E_2 is the energy of single molecules and E_{bsse} is the energy of BSSE correction.

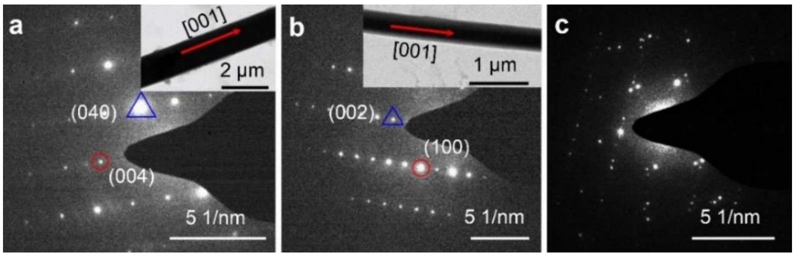

Supplementary Figure 3. (a) The SAED pattern of one typical 1D DPEpe-F_4DIB nanowire; scale bar: 5 1/nm. Inset: the TEM image of this corresponding nanowire with a scale bar of 2 μm. (b) The SAED pattern of one typical 1D DPEpe-BrFTA nanowire; scale bar: 5 1/nm. Inset: the TEM image of this corresponding nanowire with scale bar of 1 μm. (c) The SAED pattern of the heterojunction corresponding to the Figure 2b; scale bar: 5 1/nm.

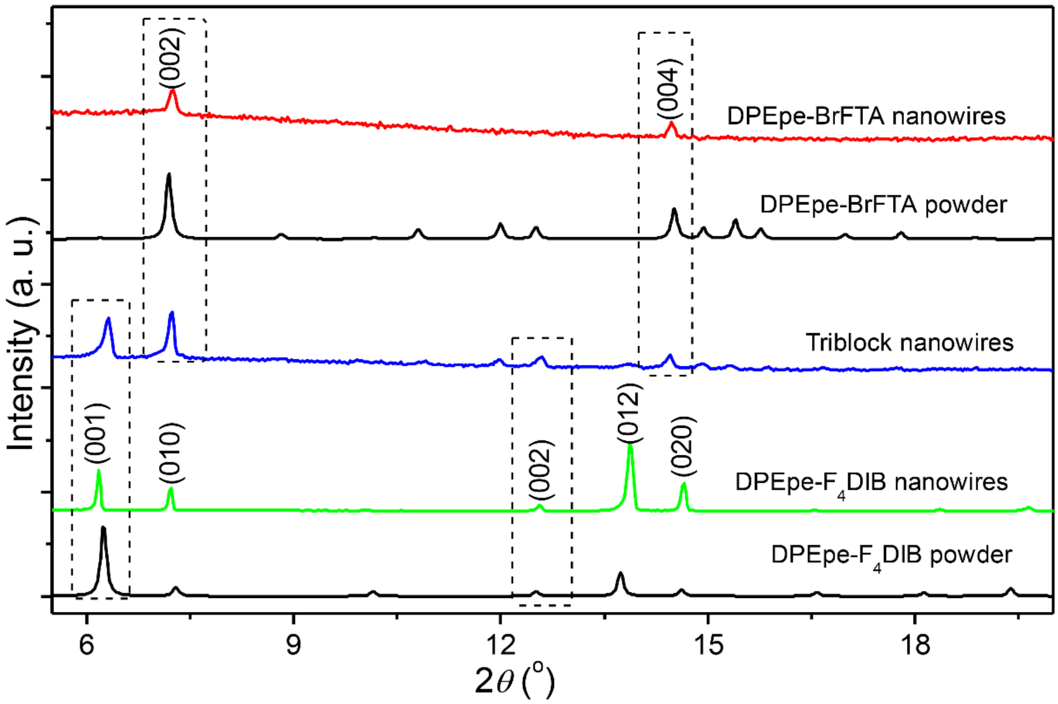

Supplementary Figure 4. X-ray diffraction patterns of the DPEpe-F_4DIB nanowires (green line), the DPEpe-BrFTA nanowires (red line), the heterostructure nanowires (blue line), and the simulated powder patterns for both PEpe-F_4DIB nanowires (down blackline) and DPEpe-BrFTA nanowires (top blackline) using the Mercury software.

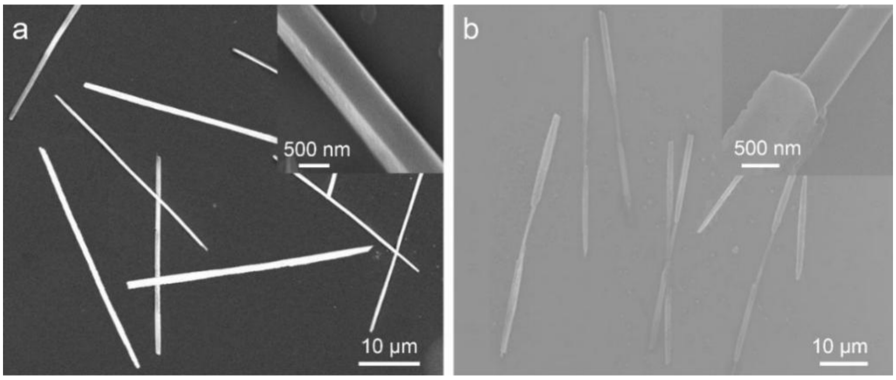

Supplementary Figure 5. The SEM images of (a) the organic triblock heterostructure nanowires and (b) the organic core/shell heterostructure nanowires. Inset: a magnified SEM image of the body of one typical organic heterostructures. The scale bars are 10 μm.

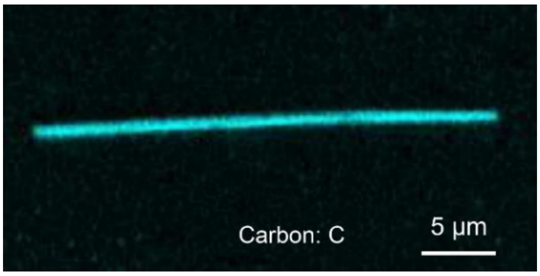

Supplementary Figure 6. The energy-dispersive X-ray spectroscopy (EDS) mapping for elements carbon (C) corresponding to the nanowires in Figure 2c. The scale bar is 5 μm.

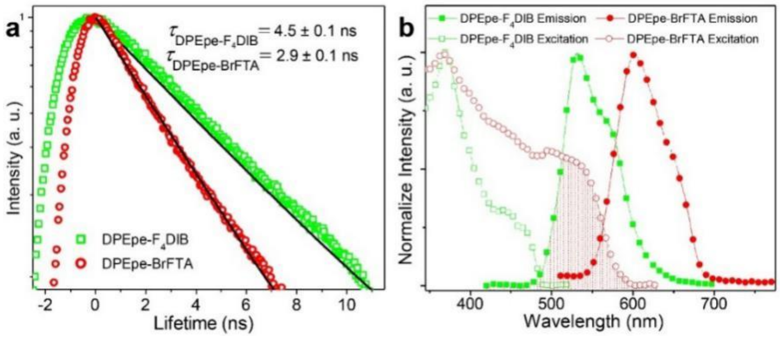

Supplementary Figure 7. (a) Time-resolved PL decay transient of the DPEpe-F_4DIB nanowires (green squares), the DPEpe-BrFTA nanowires (red circles). (b) Absorption spectra of DPEpe-F_4DIB nanowires (green hollow squares) and the DPEpe-BrFTA nanowires (red hollow circles), absorption spectra of DPEpe-F_4DIB nanowires (green solid squares) and the DPEpe-BrFTA nanowires (red solid circles).

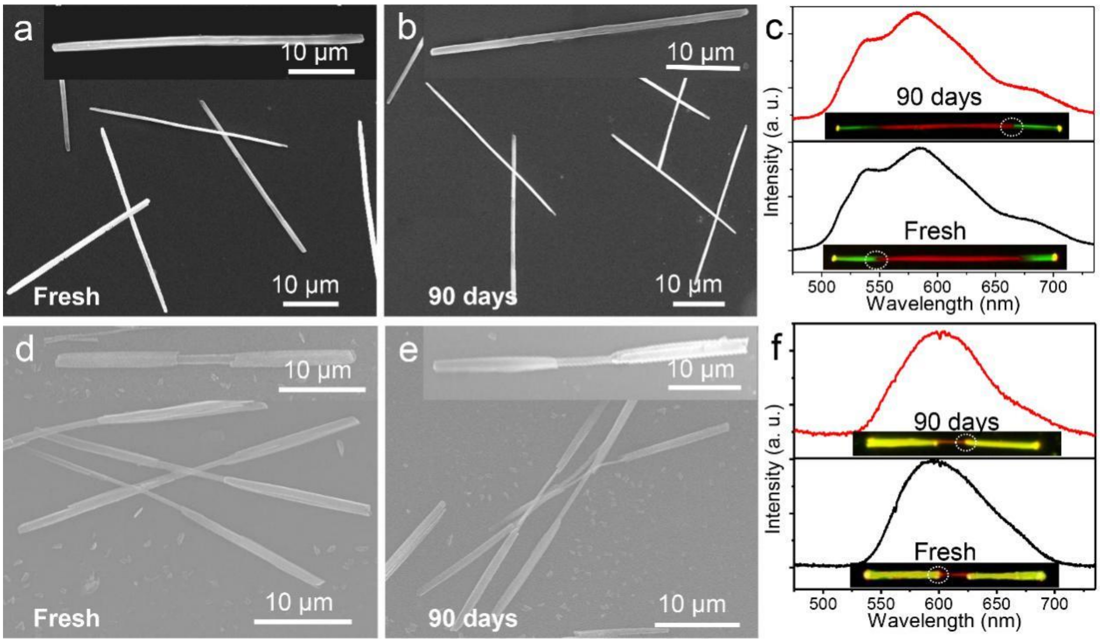

Supplementary Figure 8. The SEM images of the organic triblock heterostructure nanowires prepared (a) fresh and before (b) 90 days. Inset: the corresponding typical nanowires. The scale bars are all 10 µm. (c) The PL spectrum for the organic triblock heterostructure nanowires prepared fresh (bottom panel) and before 90 days (top panel). Inset: the corresponding FM images. The SEM images of the organic core/shell heterostructure nanowires prepared (d) fresh and before (e) 90 days. Inset: the corresponding typical nanowires. The scale bars are 10 µm. (f) The PL spectrum for the organic core/shell heterostructure nanowires prepared fresh and before 90 days. Inset: the corresponding FM images.

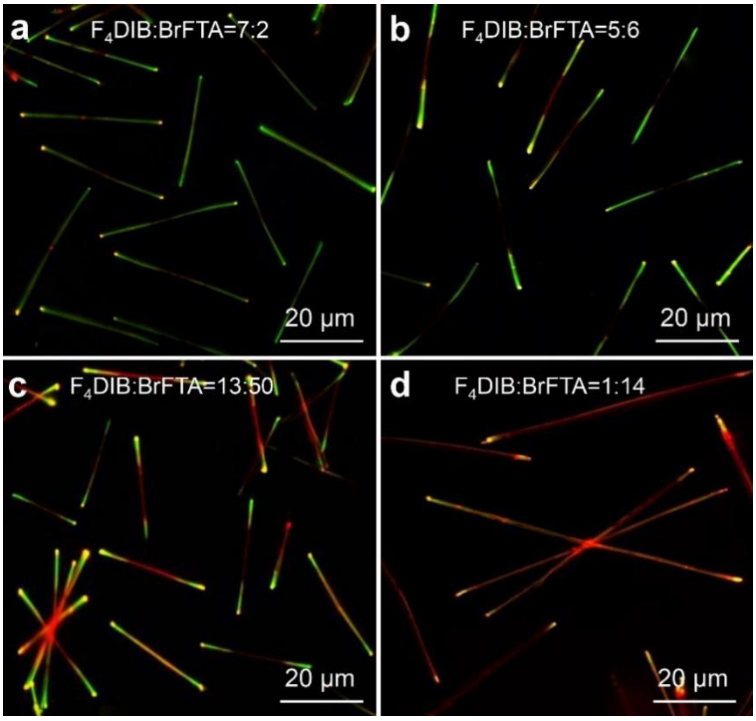

Supplementary Figure 9. FM images of G-R-G TNWs excited with the UV band (330-380 nm) based on different ratio between the F_4DIB and BrFTA: (a) 7:2, (b) 5:6, (c) 13:50 and (d) 1:14, scale bars: 20 μm.

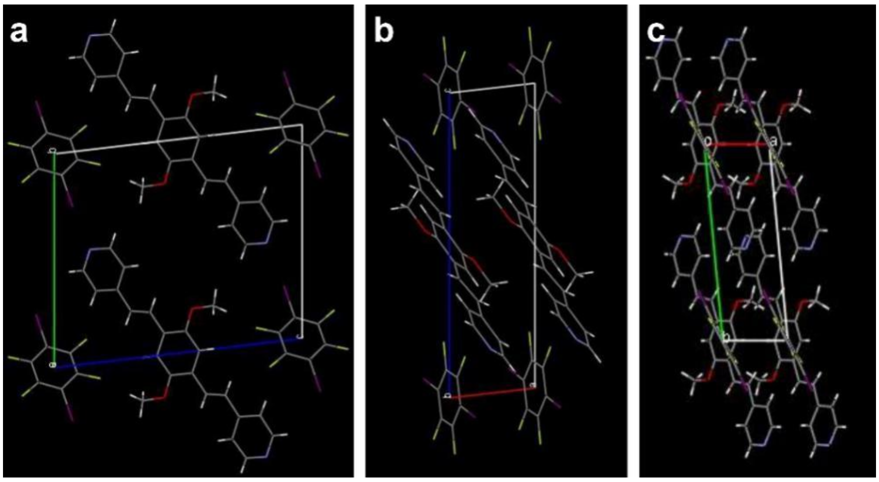

Supplementary Figure 10. The molecular packing structures of DPEpe-F_4DIB cocrystal of (a) the (100) plane, (b) the (010) plane and (c) the (001) plane.

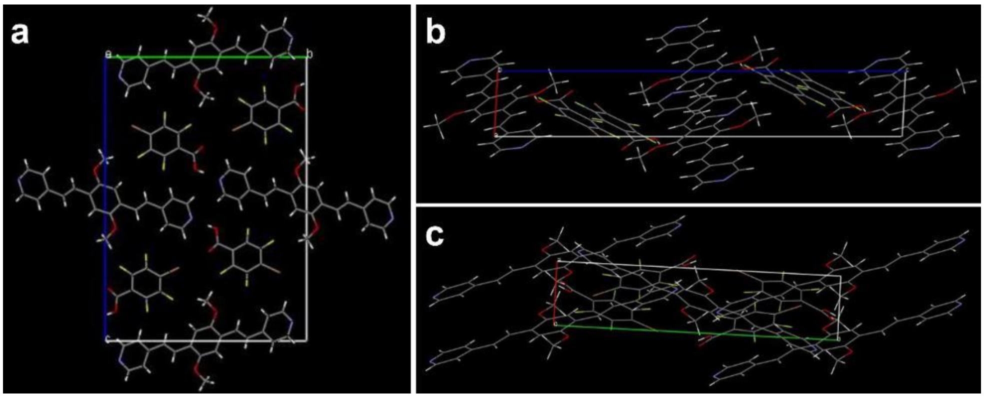

Supplementary Figure 11. The molecular packing structures of DPEpe-BrFTA cocrystal of (a) the (100) plane, (b) the (010) plane and (c) the (001) plane.

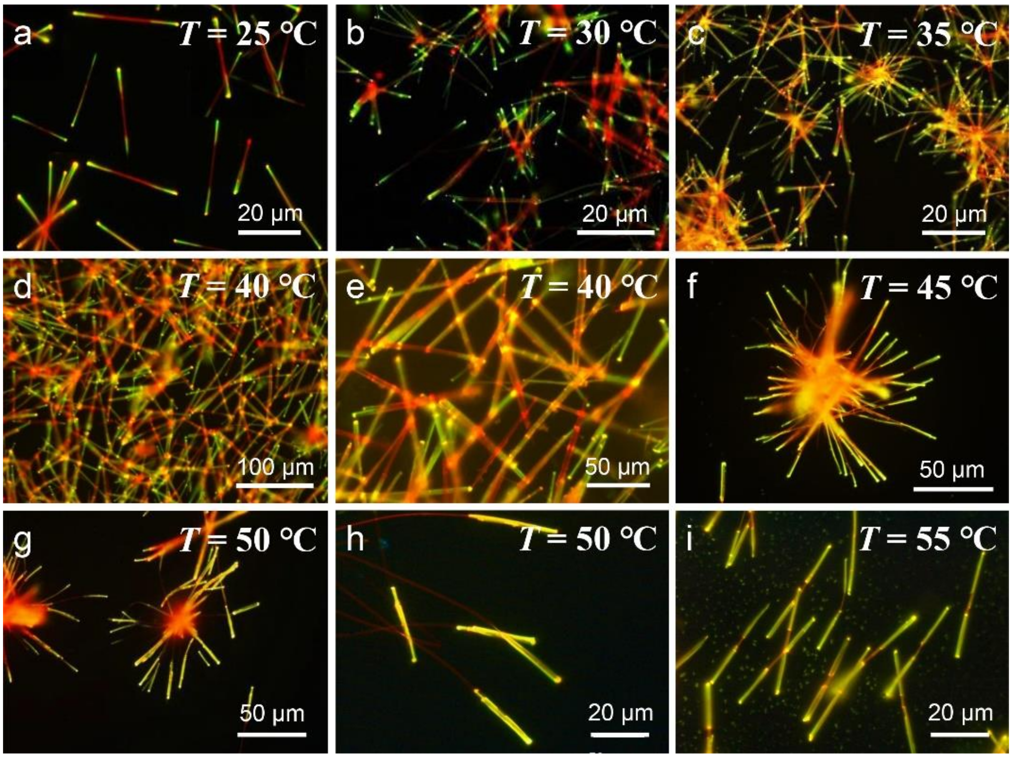

Supplementary Figure 12. FM images of 1D heterostructure nanowires based on the halogen bond and the hydrogen bond through the solution-evaporation method with stock solution at different temperatures: (a) 25 °C, (b) 30 °C, (c) 35 °C, (d-e) 40 °C, (f) 45 °C, (g-h) 50 °C, and (i) 55 °C.

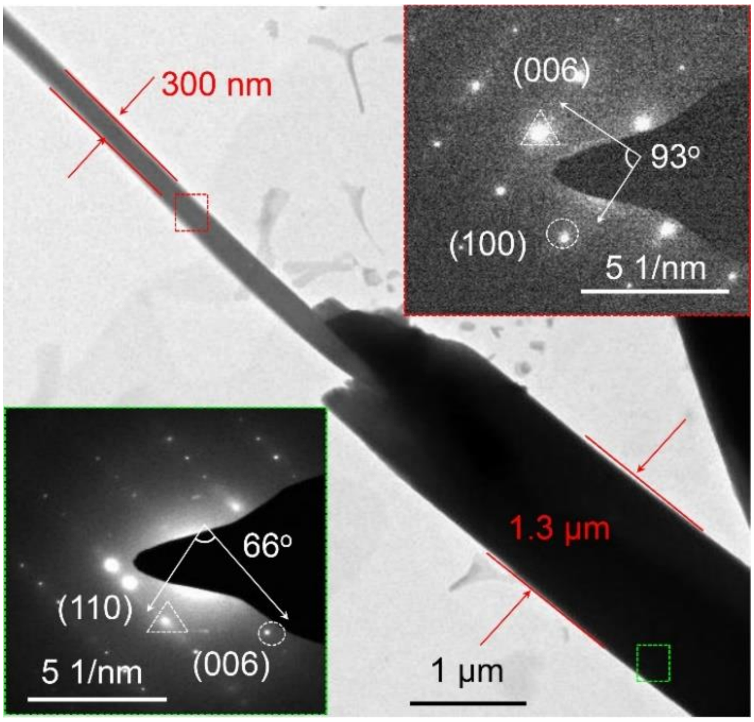

Supplementary Figure 13. The TEM image of one typical C/S-C-C/S TNW, scale bar: 1 μm. The SAED patterns at the upper-right (below-left) inset are corresponding to shell with green marked area (core red marked area) in TNWs.

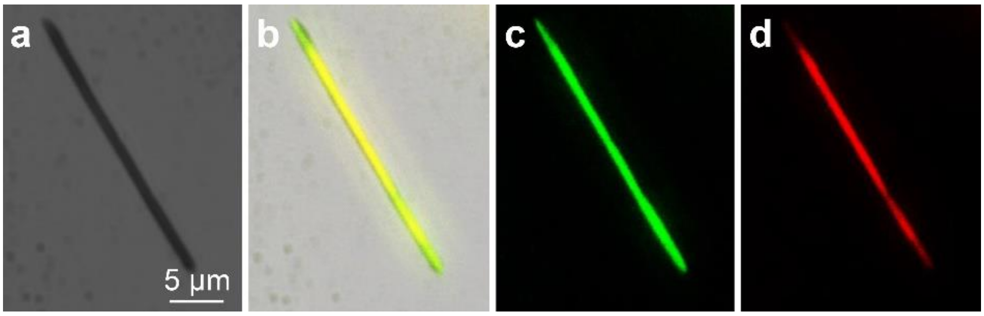

Supplementary Figure 14. Confocal laser scanning microscopy image of an individual core/shell nanowires, scale bar: 5 μm. (a) Optical image, (b) excitation at 488.0 and 543.5 nm with observation of both green and red fluorescence, (c) excitation at 488.0 nm and observation of green fluorescence and (d) excitation at 543.5 nm and observation of red fluorescence.

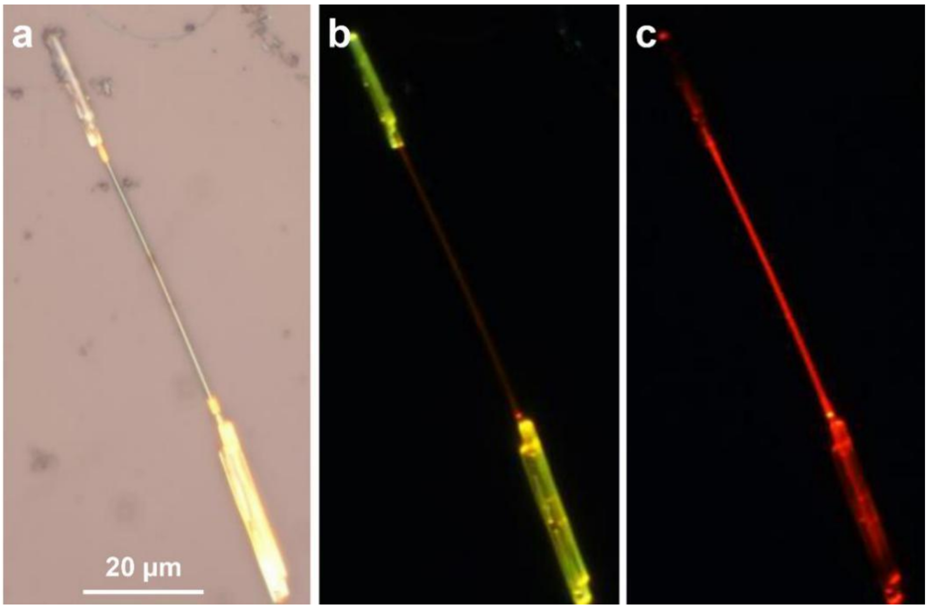

Supplementary Figure 15. (a) Optical image and corresponding FM images under excitation by (b) the UV band (330~380 nm) and (c) green-light (λ = 500~550 nm) of an individual C/S-C-C/S TNW with the ratio between F_4DIB and BrFTA of 1:3, scale bar: 20 μm.

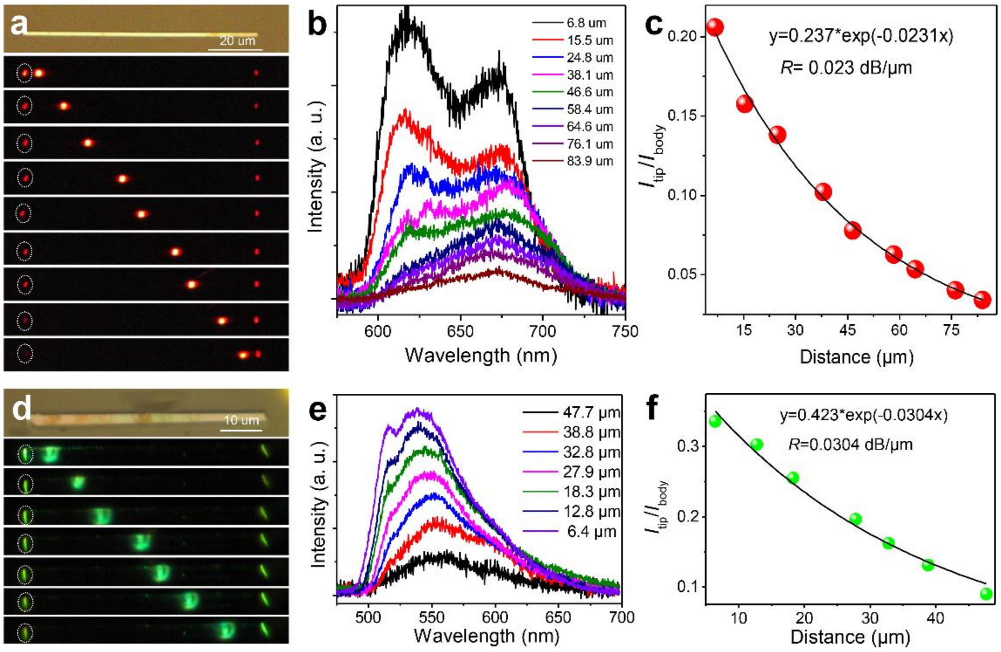

Supplementary Figure 16. (a) FM images obtained from an individual 1D DPEpe-BrFTA cocrystal by exciting with a laser beam (λ = 375 nm) at different positions with the scale bar of 20 μm. (b) The corresponding spatially resolved PL spectra in (a) with different separation distances. (c) The ratios of the intensity $I_{\text{tip}}/I_{\text{body}}$ against the distance D corresponding to (a). Curves were fitted by an exponential decay function $I_{\text{tip}}/I_{\text{body}} = A\exp(-RD)$. (d) FM images obtained from an individual 1D DPEpe-BrFTA cocrystal by exciting with a laser beam (λ = 375 nm) at different positions with the scale bar of 20 μm. (e) The corresponding spatially resolved PL spectra in (c) with different separation distances. (f) The ratios of the intensity $I_{\text{tip}}/I_{\text{body}}$ against the distance D corresponding to (c).

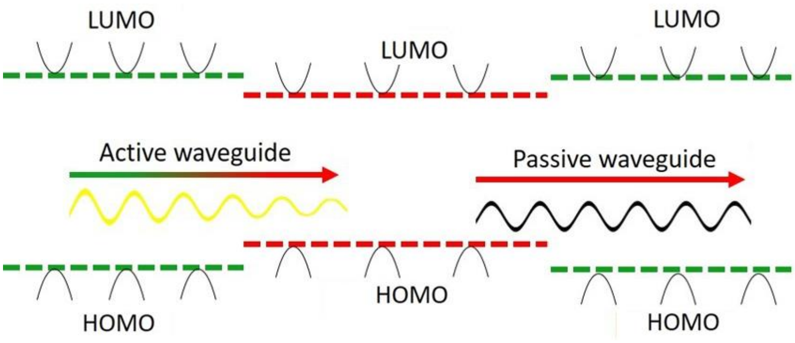

Supplementary Figure 17. Schematic illustration of the active and passive waveguides.

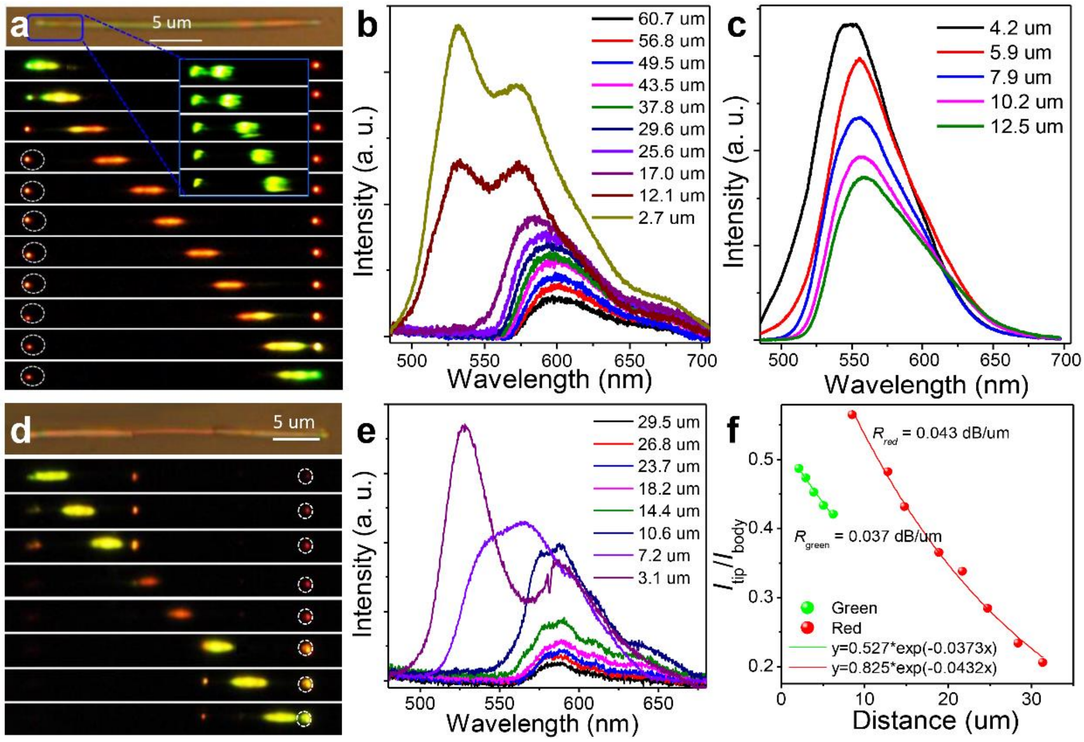

Supplementary Figure 18. (a) FM images obtained from an individual 1D G-R-G TNWs by exciting with a laser beam (λ = 375 nm) at different positions with the scale bar of 5 µm. (b and c) The corresponding spatially resolved PL spectra for (b) red and (c) green emission of the emitting tip in (a) with different separation distances. (d) FM images obtained from an individual 1DC/S-C-C/S TNWs by exciting with a laser beam (λ = 375 nm) at different positions with the scale bar of 20 µm. (e) The corresponding spatially resolved PL spectra in (d) with different separation distances. (f) The ratios of the intensity I_{tip}/I_{body} against the distance D corresponding to (a).

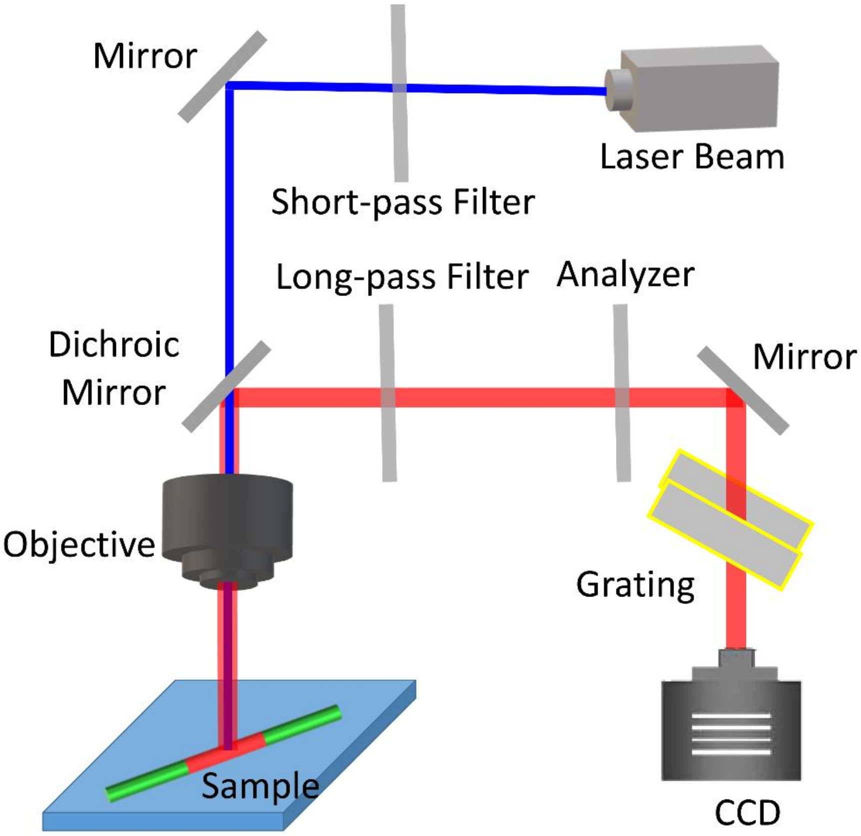

Supplementary Figure 19. Schematic demonstration of the experimental setup for the optical characterization. PL microscopy images were taken with an inverted microscope (Olympus, BX43). To measure the PL spectra of the organic heterostructure nanowires, the samples were excited locally with a 375 nm laser with a focused beam with a diameter of ~2 μm through an objective (Nikon CFLU Plan, 50×, N.A. = 0.8). The power at the input was altered by the neutral density filters. The polarization of the light at the output was altered by the analyzer. The emission from the tip of the organic heterostructure nanowires was dispersed with a grating (150 G/mm) and recorded with a thermal-electrically cooled CCD (Princeton Instruments, PIX-256E).

Supplementary Tables

Supplementary Table 1. The ratio between halogen bond acceptor (F_4DIB) and hydrogen bond acceptor (BrFTA).

Mole (mmol)			$n_{BrFTA}/2n_{DPEpe}$
DPEpe	F_4DIB	BrFTA	(%)
1	1	0	0
1	0.875	0.25	12.5
1	0.75	0.5	25
1	0.625	0.75	37.5
1	0.5	1	50
1	0.325	1.25	62.5
1	0.25	1.5	75
1	0.125	1.75	87.5
1	0	2	100

Supplementary Table 2. Crystal data and structure refinement for DPEpe-F_4DIB (CCDC No. 1838524) and DPEpe-BrFTA (CCDC No. 1892166).

Name	DPEpe-F_4DIB	DPEpe-BrFTA
Empirical formula	$C_{28}H_{20}F_4I_2N_2O_2$	$C_{36}H_{21}Br_2F_8N_2O_6$
Formula weight	746.26	889.37
Temperature	173(2) K	173(2) K
Wavelength	0.71073 Å	0.71073 Å
Crystal system	Triclinic	Monoclinic
Space Group	*P*-1	*P*21/n
Cell Lengths (Å)	a = 4.0465(2),	*a = 3.8835(2)*
	b = 12.3742(6)	*b = 17.2064(9)*
	c = 14.4568(8)	*c = 24.2582(14)*
Cell Angle (°)	α = 82.585(2),	*α = 90,*
	β = 82.769(2),	*β = 92.962(2),*
	γ = 83.758(2)	*γ = 90*
Cell Volume (Å^3)	719.22	1618.79(15)
Z: 4 *Z'*: 0	*Z*: 1 *Z'*: 0	2
R-Factor (%)	1.62	1.05

Supplementary Table 3. Interplanar crystal spacing of Various Crystal Facets {*hkl*}s in both DPEpe-F_4DIB and DPEpe-BrFTA cocrystals, respectively.

{*hkl*}s	$d_{\{hkl\}s}$ (Å)	
	DPEpe-F_4DIB	DPEpe-BrFTA
{100}s	4.06	3.88
{001}s	14.20	24.26
{012}s	6.53	9.90
{101}s	4.04	3.80
{110}s	3.96	3.78
{111}s	3.99	3.71
{011}s	9.94	14.02
{10-1}	4.04	3.86

Supplementary Table 4. Surface Free ($E^{surface}_{\{hkl\}s}$)and Attachment ($E^{attach}_{\{hkl\}s}$) Energies of Various Crystal Facets {*hkl*}s Calculated by Using the Material Studio Package.

{*hkl*}s	$d_{\{hkl\}}$ (Å)	E_{attach} (kcal mol^{-1})	$E_{surface}$ (kcal mol^{-1})
(002)s	12.1	-34.5	8.1
(011)s	14.0	-28.0	26.2
(101)s	3.7	-122.1	85.8
(11-2)s	3.7	-125.1	82.4
(11-3)s	3.5	-128.1	86.7
(113)s	3.4	-129.1	105.9
(12-1)s	3.5	-127.1	97.2
(121)s	3.5	-127.0	104.3
(123)s	3.2	-130.4	115.4
(12-3)s	3.3	-131.3	104.4
(40-2)s	0.97	-148.8	161.9

Supplementary References:

1. Hanson, G. R.; Jensen, P.; McMurtrie, J.; Rintoul, L.; Micallef, A. S. Halogen bonding between an isoindoline nitroxide and 1, 4-diiodotetrafluorobenzene: new tools and tectons for self-assembling organic spin systems. *Chem. Eur. J.* **15**, 4156-4164 (2009).
2. Gao, H. Y.; Shen, Q. J.; Zhao, X. R.; Yan, X. Q.; Pang, X.; Jin, W. J. Phosphorescent co-crystal assembled by 1, 4-diiodotetrafluorobenzene with carbazole based on C–I··· π halogen bonding. *J. Mater. Chem.* **22**, 5336-5343 (2012).
3. Zhu, W.; Zheng, R.; Zhen, Y.; Yu, Z.; Dong, H.; Fu, H., Hu, W. Rational design of charge-transfer interactions in halogen-bonded co-crystals toward versatile solid-state optoelectronics. *J. Am. Chem. Soc.* **137**, 11038-11046 (2015).
4. Johnson, S. L.; Rumon, K. A. Infrared spectra of solid 1: 1 pyridine-benzoic acid complexes; the nature of the hydrogen bond as a function of the acid-base levels in the complex1. *J. Phys. Chem.* **69**, 74-86 (1965).
5. Blinc, R.; Hadži, D. Infra-red and proton magnetic resonance spectra of solid substances containing very short hydrogen bonds. *Spectrochim. Acta* **16**, 853-862 (1960).
6. Hadži, D. Far-infrared bands of some crystals with strong hydrogen bonds. *J. Chem. Phys.* **34**, 1445-1445 (1961).
7. Kagarise, R. E. Spectroscopic studies on the soaps of phenylstearic acid. I. Infrared absorption spectra and the hydrolysis of soap films. *J. Phys. Chem.* **59**, 271-277 (1955).
8. M. J. Frisch, G. W. Trucks, H. B. Schlegel, G. E. Scuseria, M. A. Robb, J. R. Cheeseman, G. Scalmani, V. Barone, B. Mennucci, G. A. Petersson, H. Nakatsuji, M. Caricato, X. Li, H. P. Hratchian, A. F. Izmaylov, J. Bloino, G. Zheng, J. L. Sonnenberg, M. Hada, M. Ehara, K. Toyota, R. Fukuda, J. Hasegawa, M. Ishida, T. Nakajima, Y. Honda, O. Kitao, H. Nakai, T. Vreven, J. A. Montgomery, J. E. P. Jr., F. Ogliaro, M. Bearpark, J. J. Heyd, E. Brothers, K. N. Kudin, V. N. Staroverov, R. Kobayashi, J. Normand, K. Raghavachari, A. Rendell, J. C. Burant, S. S. Iyengar, J. Tomasi, M. Cossi, N. Rega, J. M. Millam, M. Klene, J. E. Knox, J. B. Cross, V.

Bakken, C. Adamo, J. Jaramillo, R. Gomperts, R. E. Stratmann, O. Yazyev, A. J. Austin, R. Cammi, C. Pomelli, J. W. Ochterski, R. L. Martin, K. Morokuma, V. G. Zakrzewski, G. A. Voth, P. Salvador, J. J. Dannenberg, S. Dapprich, A. D. Daniels, Ö. Farkas, J. B. Foresman, J. V. Ortiz, J. Cioslowski and D. J. Fox, Gaussian 09 A02.

9. Hehre, W. J.; Ditchfield, R.; Pople, J. A., *J. Chem. Phys.* **56**, 2257-2261 (1972).
